# Atypia and Surface Structure of Superficial Neoplasms of the Colon Less Than 10 mm in Diameter

**DOI:** 10.1155/DTE.2.135

**Published:** 1996

**Authors:** Masatoshi Yasuda

**Affiliations:** Third Department of Internal Medicine Ohashi Hospital Toho University School of Medicine 2-17-6 Ohashi, Meguro-ku Tokyo 153 Japan

## Abstract

Histological specimens of 30 distinct adenomas and 30 distinct carcinomas were studied by image analysis to quantify nuclear size and shape. These data were used to derive a discriminant equation, which permitted the lesions to be classified into two groups based on nuclear atypia. Next, 50 superficial type tumors of the colon (34 IIa lesions, 9 IIc + IIa lesions, and 7 IIc lesions) <10 mm in longest diameter were similarly analyzed. These lesions were classified into a high atypia index group (HAI group, 14 lesions) and a low atypia index group (LAI group, 36 lesions) by the above discriminatory equation. Differences between these two groups in surface structure, marginal zone properties, and macroscopic type, assessed using a dissecting microscope, were studied. A hyperplastic shape of the tubular orifice was seen at the marginal zone in 10 lesions (27.7%) in the LAI group and 11 lesions (78.5%) in the HAI group. This difference was significant. Surface structure and macroscopic type were not correlated with the degree of atypia. The tubular density in lesions showing a hyperplastic pitted pattern at their border was 77.40, significantly higher than that for lesions without such a pattern (73.12). The contribution of various variables to surface structure, marginal zone properties, and macroscopic type was studied by discriminant analysis. A high correlation was found between marginal zone properties and tubular density. Since lesions with high nuclear atypia *tend* to have high tubular density, marginal zone properties were secondarily correlated with the level of nuclear atypia. Observation of the marginal zone properties of lesions was thus suggested to be helpful in the diagnosis of lesions with severe atypia.


*Diagnostic and Therapeutic Endoscopy*, 1996, Vol. 2, pp. 135–146
Reprints available directly from the publisher
Photocopying permitted by license only

© 1996 OPA (Overseas Publishers Association)
Amsterdam B.V. Published in The Netherlands
by Harwood Academic Publishers GmbH
Printed in Singapore

### Atypia and Surface Structure of Superficial Neoplasms of the Colon Less Than 10 mm in Diameter

MASATOSHI YASUDA

*Third Department of Internal Medicine, Toho University School of Medicine, Tokyo, Japan*

*(Received April 3, 1995; in final form August 7, 1995)*

Histological specimens of 30 distinct adenomas and 30 distinct carcinomas were studied by image analysis to quantify nuclear size and shape. These data were used to derive a discriminant equation, which permitted the lesions to be classified into two groups based on nuclear atypia. Next, 50 superficial type tumors of the colon (34 IIa lesions, 9 IIc + IIa lesions, and 7 IIc lesions) <10 mm in longest diameter were similarly analyzed. These lesions were classified into a high atypia index group (HAI group, 14 lesions) and a low atypia index group (LAI group, 36 lesions) by the above discriminatory equation. Differences between these two groups in surface structure, marginal zone properties, and macroscopic type, assessed using a dissecting microscope, were studied. A hyperplastic shape of the tubular orifice was seen at the marginal zone in 10 lesions (27.7%) in the LAI group and 11 lesions (78.5%) in the HAI group. This difference was significant. Surface structure and macroscopic type were not correlated with the degree of atypia. The tubular density in lesions showing a hyperplastic pitted pattern at their border was 77.40, significantly higher than that for lesions without such a pattern (73.12). The contribution of various variables to surface structure, marginal zone properties, and macroscopic type was studied by discriminant analysis. A high correlation was found between marginal zone properties and tubular density. Since lesions with high nuclear atypia *tend* to have high tubular density, marginal zone properties were secondarily correlated with the level of nuclear atypia. Observation of the marginal zone properties of lesions was thus suggested to be helpful in the diagnosis of lesions with severe atypia.

**KEY WORDS:** Image analysis, nuclear atypia, dissecting microscope, superficial neoplasms of colon

### INTRODUCTION

The detection of small superficial type neoplasms of the colon has increased recently. Included among these lesions are many small (longest diameter <10 mm), rapidly progressive carcinomas that invade the submucosa. Clinically, the accurate diagnosis of these lesions has become an urgent task.[1-3] The atypia of small lesions that invade the submucosa has been reported to be severe, both in the mucosa and the submucosa.[4] Clinical identification of small lesions with severe atypia requires an understanding of their macroscopic characteristics. Numerous studies have addressed this issue by investigating the relationship between pathological diagnosis and the superficial characteristics of lesions. However, the lack of consistent pathological criteria for the diagnosis of intramucosal lesions calls into question the credibility of previously reported results. Further problems are associated with significant interlaboratory differences in the assessment of atypia for depressed type lesions. These factors have precluded the development and acceptance of objective criteria defining the superficial characteristics of carcinomas.

The initial aim of this study was to develop a discriminatory equation that would differentiate colonic carcinomas from adenomas based on nuclear atypia as assessed by image analysis. This was accomplished by studying a series of clear-cut colonic carcinomas that invaded the submucosa or deeper and a series of adenomas as controls. The resulting discriminant equation was then applied to superficial type neoplasms of the colon (longest diameter <10 mm) that were resected endoscopically. The diagnosis derived by this discriminant equation was then compared with the histopathological diagnosis. The cor-

Address for correspondence: Masatoshi Yasuda, M.D., Third Department of Internal Medicine, Ohashi Hospital, Toho University School of Medicine, 2-17-6 Ohashi, Meguro-ku, Tokyo 153, Japan.

relation with macroscopic type, surface properties, and marginal zone properties was assessed using a dissecting microscope, and was studied to identify objective variables that would facilitate diagnosis.

### SUBJECTS AND METHODS

As controls, 30 surgically resected colonic carcinomas invading to the submucosa or deeper (11 sm carcinomas, 8 pm carcinomas, 11 ss carcinomas) and 30 endoscopically resected pedunculated or semipedunculated colonic adenomas showing mild atypia were studied. All lesions were diagnosed in this department between April 1991 and September 1993. All carcinomas were well differentiated adenocarcinomas, and the region of submucosal invasion was studied. The adenomas showed mild atypia and satisfied all of the criteria proposed by Watanabe et al.[5] for colonic adenomas.

Micrographs of the designated areas in pathological specimens in the control group were displayed on a monitor screen connected to a computer. The measurements described below were then made using a manually operated image analyzer (Rise Co., type EM).

Atypia was quantified by measuring nuclear area, longest diameter, and shortest diameter as indices of nuclear size. The nuclear longest shortest diameter ratio and the shape index were measured as indices of nuclear shape. The shape index was calculated by the formula: nuclear circumference$^2$/nuclear area $\times$ $1/4\pi$. This value expresses the degree of divergence from a round shape. On the monitor, nuclei of tubule cells, considered to most closely reflect atypia, were measured in groups of 50 at a magnification of 2500×, and measurements were expressed as mean values. Any overlapping nuclei or nuclei with indistinct margins were excluded.

The atypia indices that significantly differed between the control carcinomas and adenomas underwent discriminatory analysis to derive an equation that would differentiate these lesions.

Next, 50 small (longest diameter <10 mm), superficial type neoplasms of the colon, obtained during the study period by endoscopic resection, were studied. Histopathologically, lesion height was less than twice the thickness of the surrounding normal mucosa and growth was primarily horizontal, creating the appearance of a flat lesion. Hemispherical lesions were excluded. The surface structure of all neoplasms was examined using a dissecting microscope. All lesions also underwent careful histological examination, and the grade of atypia within a given lesion was regarded to be histologically homogeneous. Included in this series were 6 mucosal carcinomas, 10 adenomas with severe atypia, 32 adenomas with moderate atypia, and 2 adenomas with mild atypia. The macroscopic type according to the classification of the Japanese Research Society for Cancer of the Colon and Rectum, was IIa 34 lesions, IIc + IIa 9 lesions, and IIc 7 lesions. Macroscopic type was determined based primarily on the shape in histological sections. Endoscopic findings were also referred to.

Endoscopically resected lesions were spread out on pieces of rubber using a pin, taking care to avoid excessive distention, and immediately fixed in 10% formalin solution. After 24 hours, any surface mucus was removed by washing thoroughly with tap water. The lesions were stained with Carrazi's hematoxylin stain, and the surface structure and marginal zone were examined at a magnification of 7.5 to 40× using a dissecting microscope (Olympus, model SZH).

The central surface structure of the lesions was classified based on the morphology of the orifice of tubules into IIIS type and IIIL type as described by Kudo et al.[6] The configuration of the marginal zone was classified as A type (Fig. 1A), showing a sharp boundary with normal surrounding crypts, or B type (Fig. 1B), showing an irregular wavy pattern with hyperplastic surrounding crypts, referring to the classification of Tada et al.[7]

In addition to the aforementioned observations, tubular density was measured according to the method described by Nakamura.[4] Regions with relatively uniform tubular density were magnified 450× and projected on a monitor. Tubular density was calculated by the formula: tubular density = total tubule area per 20 × 8 cm (corresponding to 444 × 178 μm)/20 × 8 cm. For each lesion, tubular densities at a total of five different sites were calculated, and the mean value was used as the tubular density of the lesion.

Using the discriminatory equation obtained from the control group ($Z = 15.311 + 0.285 \times$ nuclear area $- 10.497 \times$ nuclear longest/shortest diameter ratio), the lesions were divided into two groups: one showing a high atypia index (HAI) of $Z > 0$, and the other showing a low atypia index (LAI) of $Z < 0$.

The resulting HAI group and LAI group were studied to identify differences in macroscopic type and surface and marginal zone properties, examined with a dissecting microscope. Differences in ductal density between the groups were also investigated.

Macroscopic type, central surface properties, and marginal zone properties were contrasted with the aforementioned nuclear measurements and tubular density. Based on macroscopic type the lesions were divided into two groups [protruding type lesions (IIa) and lesions showing depression (IIc and IIc + IIa)], and discriminant analysis

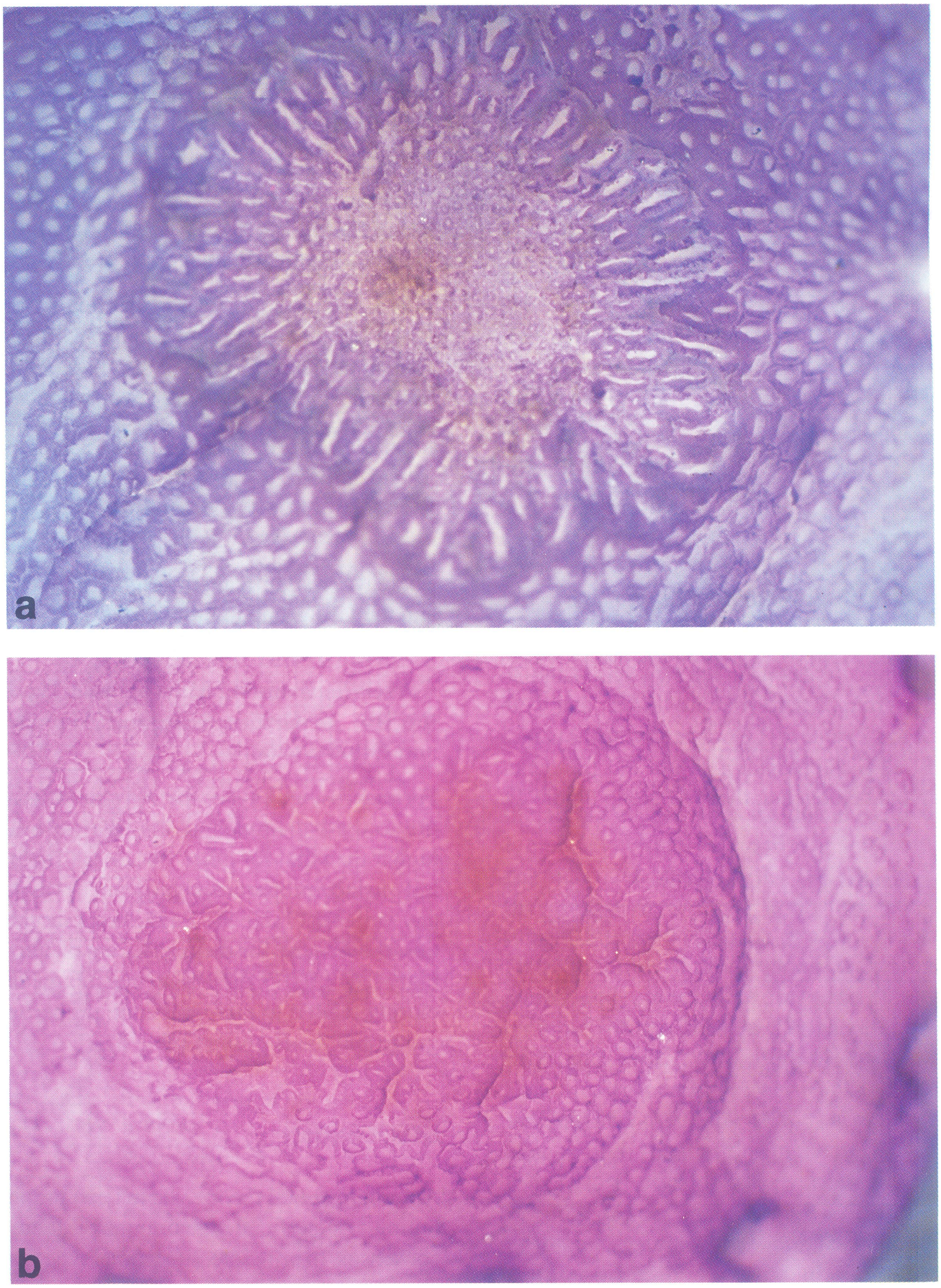

**Figure 1** A. A type marginal zone properties showing a sharp boundary with the normal surrounding tubules. B. B type marginal zone properties showing an irregular wavy pattern with hyperplastic surrounding tubules.

was performed using the measured variables (nuclear area, longest diameter, shortest diameter, longest/shortest diameter ratio, shape index, and tubular density). The significance (*F* value and probability) of the coefficient corresponding to each of the variables of the resulting linear discriminant equation was determined. Discriminant analyses were likewise performed for surface and marginal zone properties, and the significance of the coefficient for each variable was determined. For statistical analysis, the Mann-Whitney test and $\chi^2$ test were used. Differences with a probability of $p < 0.05$ were regarded to be significant.

### RESULTS

### Nuclear Measurement in Control Group

Carcinomas and adenomas differed significantly in regard to nuclear area, longest diameter, shortest diameter, longest/shortest diameter ratio, and shape index ($p < 0.1$) (Table 1). Among these five variables, discriminant analysis using 2 or more variables may be performed 21 ways. The formula that yielded the highest accuracy with the fewest variables and the lowest error discrimination rate was determined. When discriminant analysis was performed using the two variables of nuclear area and longest/shortest diameter ratio, the accuracy of the resulting equation for the 60 lesions in the control group was 100%, and the error discrimination rate, assuming that the pathological diagnosis was correct, was 4.4%, which was the lowest value obtained.

### Atypia Index and Histopathological Diagnosis

The 50 lesions in the study group were classified by discriminant analysis into 14 lesions in the HAI group and 36 lesions in the LAI group. There were no lesions that were unclassifiable due to a $Z$ value of 0. Compared with the pathological diagnosis, all 6 carcinomas and 8 of the 10 adenomas with high atypia belonged to the HAI group (Table 2).

**Table 1** Nuclear Measurement in the Control Group

| | *Distinct Carcinomas (n = 30)* | *Distinct Adenomas (n = 30)* |
|---|---|---|
| Nuclear area ($\mu m^2$) | 49.70 ± 12.76* | 23.25 ± 5.05 |
| Nuclear longest diameter ($\mu m$) | 10.80 ± 1.57* | 9.21 ± 0.99 |
| Nuclear shortest diameter ($\mu m$) | 5.94 ± 0.71* | 3.25 ± 0.45 |
| Nuclear shortest/longest diameter ratio | 1.89 ± 0.24* | 3.00 ± 0.39 |
| Shape index† | 1.28 ± 0.24* | 1.64 ± 0.14 |

*$p < 0.001$.
†Shape index = nuclear circumference²/nuclear area × 1/4 π.

**Table 2** Relationship between nuclear Atypia Index and Histopathological Diagnosis*

| | | *Adenomas with* | | |
|---|---|---|---|---|
| | *Carcinomas* | *Severe Atypia* | *Moderate Atypia* | *Mild Atypia* |
| HAI ($n = 14$) | 6 | 8 | 0 | 0 |
| LAI ($n = 36$) | 0 | 2 | 32 | 2 |

*Discriminant equation: $Z = 15.311 + 0.285 \times$ [nuclear area] − $10.497 \times$ [nuclear shortest/longest diameter ratio]. $Z > 0$: HAI; $Z < 0$: LAI.

### Relationship between Atypia Index and Macroscopic Type

There was no difference in macroscopic type between the HAI group and the LAI group. Three IIc lesions and 5 IIc + IIa lesions were included in the LAI group (Table 3).

### Relationship between Nuclear Atypia Index and Central Surface Properties

There was no significant difference between the HAI group and the LAI group. Analysis of the relationship between nuclear atypia index and marginal zone properties revealed significantly more lesions with a B-type marginal zone in the HAI group (Table 4).

### Relationship between Nuclear Atypia Index and Tubular Density

There was a significant difference between the HAI group and the LAI group in tubular density ($p < 0.05$) (Table 5).

### Correlations among Macroscopic Type, Structure of the Tubular Orifice, Marginal Zone Properties, and Other Variables

For macroscopic type, the $F$ value of the coefficient for the longest diameter of the nucleus was significantly higher than that for other variables (Table 6), but for central surface properties significance was not obtained for any variable (Table 7). Regarding marginal zone properties, the $F$ value for the coefficient of tubular density was highest ($p < 0.01$), indicating that tubular density was most closely related to marginal zone properties in both the A group and the B group (Table 8).

### DISCUSSION

Although several theories have been proposed for the definition of superficial type tumors,[9–13] there is still a lack of consensus. In principle, superficial type tumors are defined according to the criteria for early gastric cancer. Macroscopically, these lesions appear as slightly protruding or depressed lesions. However, as the structure of low,

**Table 3** Relationship between Atypia Index and Macroscopic Type

| | *IIa* | | *IIc + IIa* | | *IIc* | |
|---|---|---|---|---|---|---|
| HAI ($n = 14$) | 6 | NS | 4 | NS | 4 | NS |
| LAI ($n = 36$) | 28 | | 5 | | 3 | |

small lesions may appear to change with the degree of insufflation applied during endoscopy, it is often difficult to differentiate between I and IIa lesions or IIc and IIc + IIa lesions. Histologically, superficial type lesions have horizontal tubules. Lesions higher than the surrounding normal mucosa are designated as superficial protruding type (IIa or IIa + IIc). Those at the same level as the surrounding mucosa are designated as superficial flat type (IIb), and lesions that are lower than the surrounding mucosa as superficial depressed type (IIc).[13] Classification may be difficult because pathological sections may not be correctly prepared into longitudinal sections. Lesions with a height that did not exceed the thickness of the normal surrounding mucosa, as evaluated in pathological sections, were regarded as superficial type. Histological characteristics were contrasted with endoscopic and dissecting microscopic findings, and protruding lesions with a horizontal head were defined as pure IIa type. Any lesions with a slightly protruding structure were excluded from this study.

Since Kudot et al.[6] emphasized the clinical importance of depressed type carcinomas of the colon, increasing numbers of reports on these lesions have appeared in the literature. However, distinctly protruding type lesions are frequently misclassified as depressed type due to the presence of a slight surface depression, creating considerable confusion. Therefore, in this study superficial depressed type was used only to designate superficial type lesions that were predominantly depressed in pathological specimens. Lesions that were predominantly protruding with a shallow depression on their surface were classified as IIa. Superficial depressed lesions are frequently associated with protrusion around the lesion. However, only lesions showing reactive protrusion without histopathological signs of neoplastic tubules at the surrounding protrusion were regarded as IIc. Lesions with tubules in the surrounding protrusion were classified as IIc + IIa (Figs. 2 and 3).

Increased detection of superficial type neoplasms of the colon has motivated various clinical studies on these lesions. However, the reliability of previous studies has been compromised due to the lack of uniform pathological criteria for mucosal carcinomas. This can also be inferred from the considerable differences between centers in the relative frequencies of carcinomas among all colonic neoplasms as well as the rates of carcinomas with and without adenoma components.

To make the histological diagnosis of cancer more objective, Nakamura et al.[14] assigned numerical scores to the atypia of tumorous lesions at cytological and histological levels, and assessed whether lesions were benign or malignant based on the total score. They measured the nucleus/cytoplasm area ratio per tubule and the tubule/interstitial area ratio (tubular density). Determination of these variables facilitated a more objective and reproducible diagnosis of adenomas and carcinomas. Watanabe et al.,[5] however, pointed out several problems with the method of Nakamura et al. Specifically mentioned were lack of adequate regard for nuclear and nucleolar shape, a main characteristic of cancer, high fluctuations in the measured variables (i.e., ratios), and considerable variations in the results depending on specimen staining intensity. To resolve these problems, Ajioka et al.[15] emphasized the importance of cellular differentiation (cellular atypia) as an indicator

**Table 4** Relationship between Nuclear Atypia Index and Dissecting Microscopic Findings*

| | *Surface Properties* | | | *Marginal Zone Properties* | | |
|---|---|---|---|---|---|---|
| | *III S* | *III L* | | *A* | *B* | |
| HAI (*n* = 14) | 13 | 1 | NS | 3 | 11 | **$p < 0.01$ |
| LAI (*n* = 36) | 27 | 9 | | 26 | 10 | |

*III S: showing tubular or round pits smaller than normal; III L: showing tubular or shaped pits larger than normal. A: showing a sharp boundary with the normal surrounding tubules; B: showing an irregular wavy pattern with hyperplastic surrounding tubules.

**Table 5** Relationship between Nuclear Atypia Index and Tubular Density

| | *Tubular Density (%)* | |
|---|---|---|
| HAI (*n* = 14) | 77.40 ± 4.79 | $p < 0.05$ |
| LAI (*n* = 36) | 73.12 ± 5.61 | |

**Table 6** Relationship between Macroscopic Type and Each Parameter

| | *IIa (n = 34)* | *IIc or IIc + IIa (n = 16)* | *F Value (Probability)* |
|---|---|---|---|
| Nuclear area (μm²) | 33.81 ± 4.70 | 32.17 ± 6.64 | 3.59 |
| Nuclear longest diameter (μm) | 10.52 ± 1.23 | 10.32 ± 0.97 | 4.96* |
| Nuclear shortest diameter (μm) | 4.33 ± 0.41 | 4.09 ± 0.56 | 0.11 |
| Nuclear shortest longest diameter ratio | 2.55 ± 0.39 | 2.69 ± 0.32 | 1.62 |
| Shape index | 1.51 ± 0.18 | 1.57 ± 0.14 | 0.13 |
| Tubular density (%) | 75.12 ± 4.89 | 73.93 ± 6.04 | 0.04 |

*$p < 0.05$.

**Table 7** Relationship between Surface Properties and Each Variable

| | *IIIS* (n = *40)* | *IIIL* (n = *10)* | *F Value (Probability)* |
|---|---|---|---|
| Nuclear area (μm²) | 32.81 ± 6.04 | 32.22 ± 6.47 | 0.05 |
| Nuclear longest diameter (μm) | 10.37 ± 1.09 | 10.43 ± 0.94 | 3.32 |
| Nuclear shortest diameter (μm) | 4.19 ± 0.50 | 4.09 ± 0.63 | 3.13 |
| Nuclear shortest/longest diameter ratio | 2.61 ± 0.35 | 2.76 ± 0.33 | 3.36 |
| Shape index | 1.54 ± 0.15 | 1.61 ± 0.15 | 0.02 |
| Tubular density (%) | 74.38 ± 5.76 | 74.06 ± 5.56 | 0.04 |

**Table 8** Relationship between Marginal Zone and Each Variable

| | *A group (n = 29)* | *B group (n = 21)* | *F Value (Probability)* |
|---|---|---|---|
| Nuclear area (μm²) | 31.51 ± 5.13 | 34.34 ± 6.97 | 0.28 |
| Nuclear longest diameter (μm) | 10.37 ± 1.03 | 10.40 ± 1.11 | 0.17 |
| Nuclear shortest diameter (μm) | 4.00 ± 0.43 | 4.40 ± 0.57 | 0.19 |
| Nuclear shortest/longest diameter ratio | 2.74 ± 0.32 | 2.50 ± 0.35 | 0.03 |
| Shape index | 1.59 ± 0.14 | 1.50 ± 0.15 | 0.00 |
| Tubular density (%) | 70.85 ± 4.04 | 79.10 ± 4.00 | 33.46* |

* $p < 0.001$.

of the atypia of colonic carcinomas, and classified lesions as having high or low atypia based on nuclear structure.

With emphasis on differences in nuclear size and structure between clearly defined carcinomas and adenomas, in the present study nuclear size and shape were considered to be the most important factors in the diagnosis of atypia, similar to Ajioka et al. These variables were measured, expressed numerically, and used to derive a discriminant equation that classified lesions as having high-grade or low-grade atypia. Carcinomas were included in the control group only if they fulfilled the diagnostic criteria for carcinomas proposed by Morson and Dawson;[16] in addition, all control carcinomas invaded the submucosa and were consistently diagnosed as carcinomas by a group of pathologists. The study group consisted of 30 randomly selected carcinomas that invaded the submucosa. All of these lesions were surgically resected during the same period as the control lesions. The concepts of low atypia and high atypia carcinomas proposed by Watanabe et al.[5] and of polypoid and nonpolypoid growth type proposed by Shimoda et al.[17] were not considered. However, although slowly and rapidly progressing carcinomas or carcinomas with other distinct characteristics such as different grades of atypia are know to exist, atypia of carcinomas is fundamentally a continuously evolving process, and the criteria for each stage have not been established. In this study, the carcinomas in the control group were randomly selected to represent carcinomas in general. The fact that significant differences were noted in nuclear dimensions between the control adenoma group and the control carcinoma group indicates that these groups were appropriate as controls for the discriminant analysis.

Generally, after invading the submucosa, carcinomas develop differently than they do in the mucosa. It is thus inappropriate to measure the tubular density in regions of submucosal cancer invasion and directly compare it with the tubular density of intramucosal lesions. Therefore, in the present study, tubular density was excluded from discriminant analysis, and it was treated as an independent variable. As an index of atypia, the nucleus/cytoplasm ratio (the nuclear area divided by the sum of the cytoplasmic area and the nuclear area) is known to be higher in carcinomas than in tubule ducts, but as atypia increases, nuclei become less stratified, resulting in decreased nuclear area and, consequently, a smaller nucleus/cytoplasm ratio. This variable is not directly related to the atypia of carcinomas invading the submucosa. It was thus not studied.

The resulting discriminant equation was a first-order linear equation, with the nuclear area as an index of nuclear size and the nuclear longest diameter/shortest diameter ratio an index of nuclear shape. The sum of these variables serves to classify lesions into a high-atypia group and a low-atypia group. The error discrimination rate was set as low as possible statistically, and in fact the error discriminatory rate for the two control groups was 0 (accuracy 100%).

The HAI group defined by the discriminant equation was found to correspond to carcinomas and highly atypical adenomas, by comparison with actual pathological diagnoses. Strictly speaking, however, the LAI group showed some noncorrespondence because it included two highly atypical adenomas. On histological reexamination,

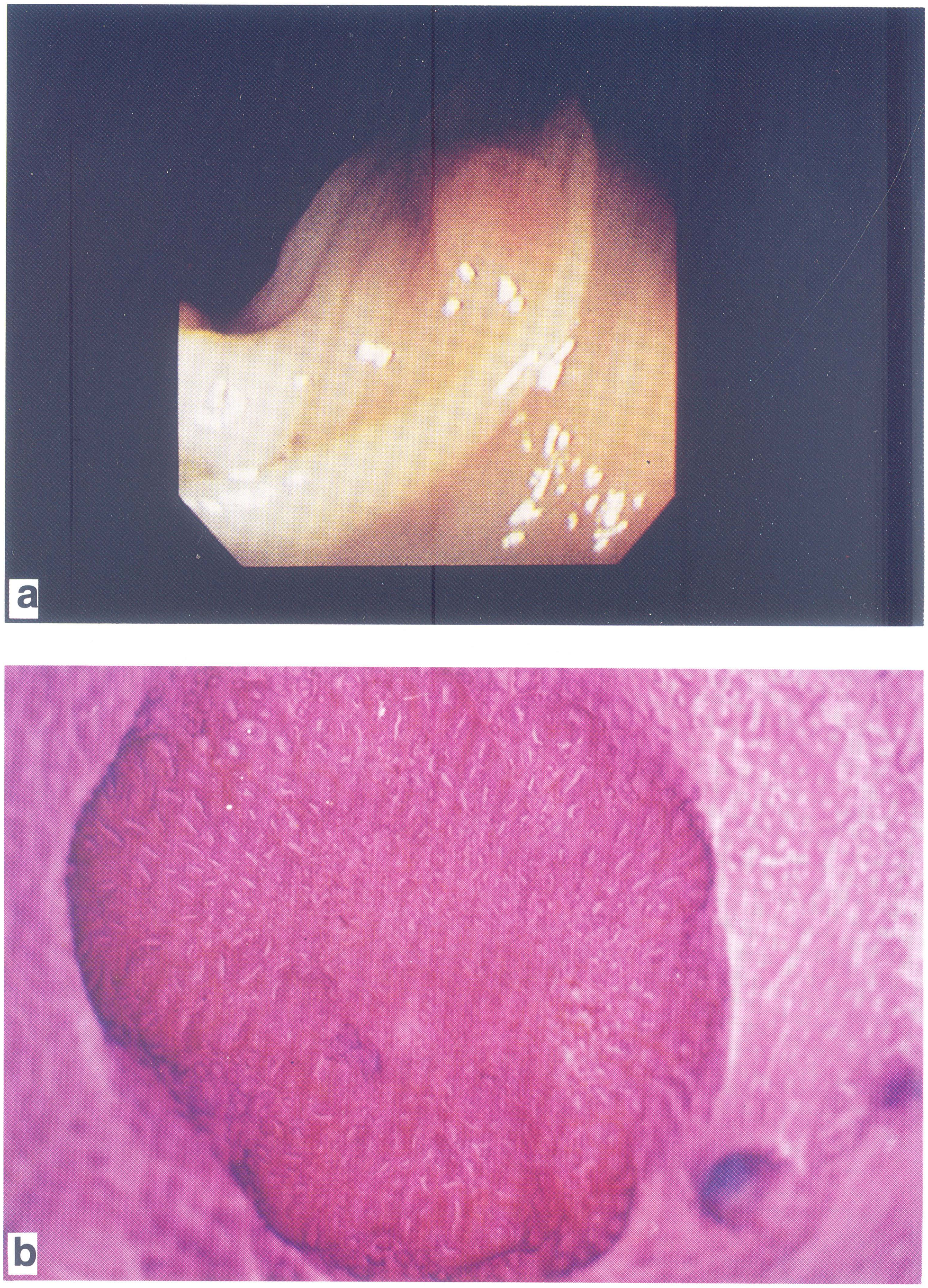

**Figure 2** (*Continued*)

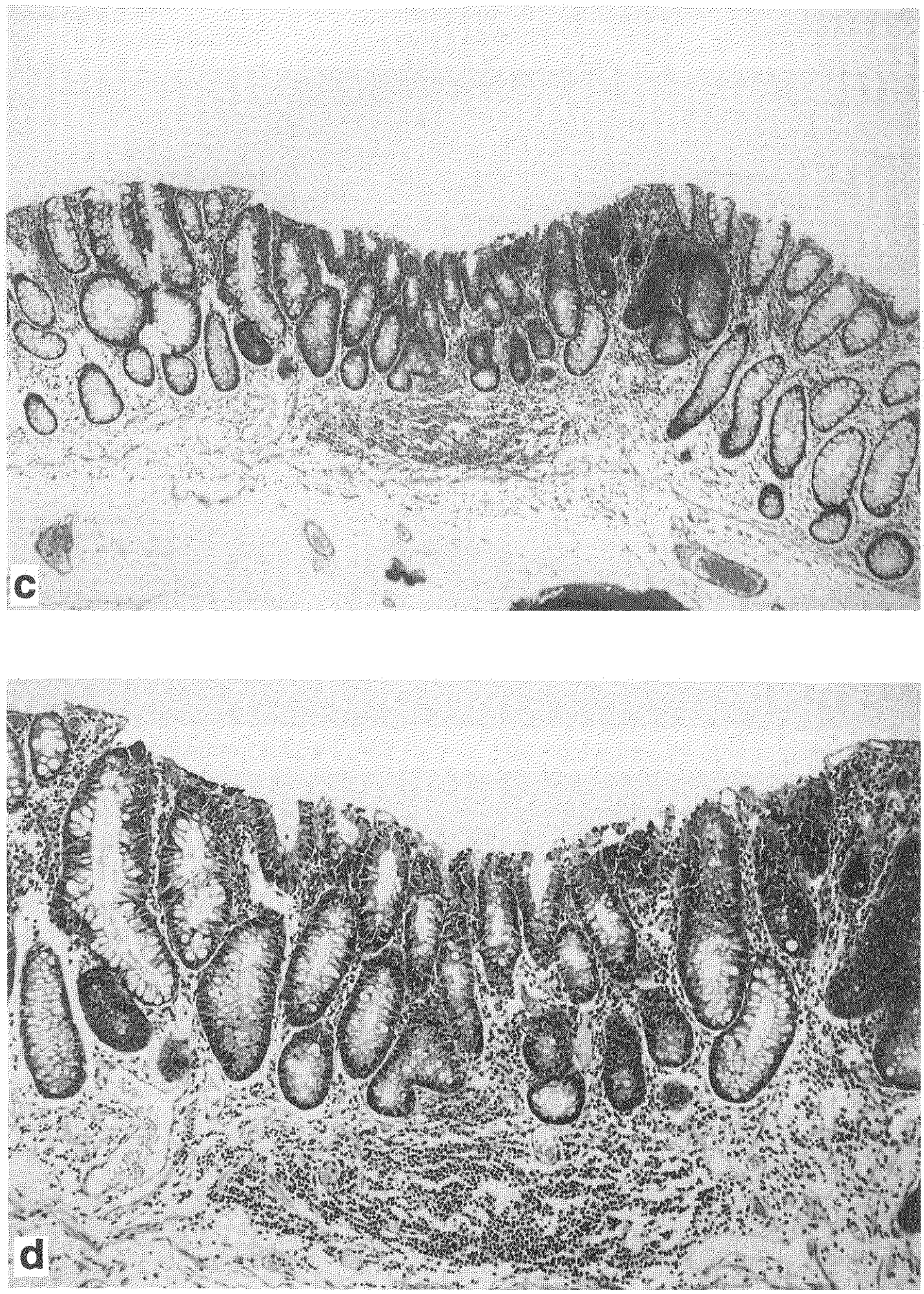

**Figure 2** A. Colonoscopic picture showing a small, flat, elevated lesion with depression in the descending colon. The depressed area was slightly hyperemic. B. Dissecting microscopic view of a lesion of 5 mm in diameter. The surface structure was IIIL at part of the periphery, but a IIIS pattern was mainly seen. Its margin was relatively sharp with normal surrounding tubules. The marginal zone was classified as A type. C. Cross-section of Figure 2B. The depressed area was lower than the normal mucosa, and neoplastic tubules were seen in the surrounding elevated area, showing IIc + IIa. D. Histological picture at low magnification. The tubular density of the lesion was 75.79%, nuclear area 30.35 $\mu m^2$, and nuclear shortest/longest diameter ratio 2.56. The atypia index obtained from the discriminatory equation was –2.91. So this lesion was classified into the LAI group. The histopathological diagnosis was adenoma with moderate atypia.

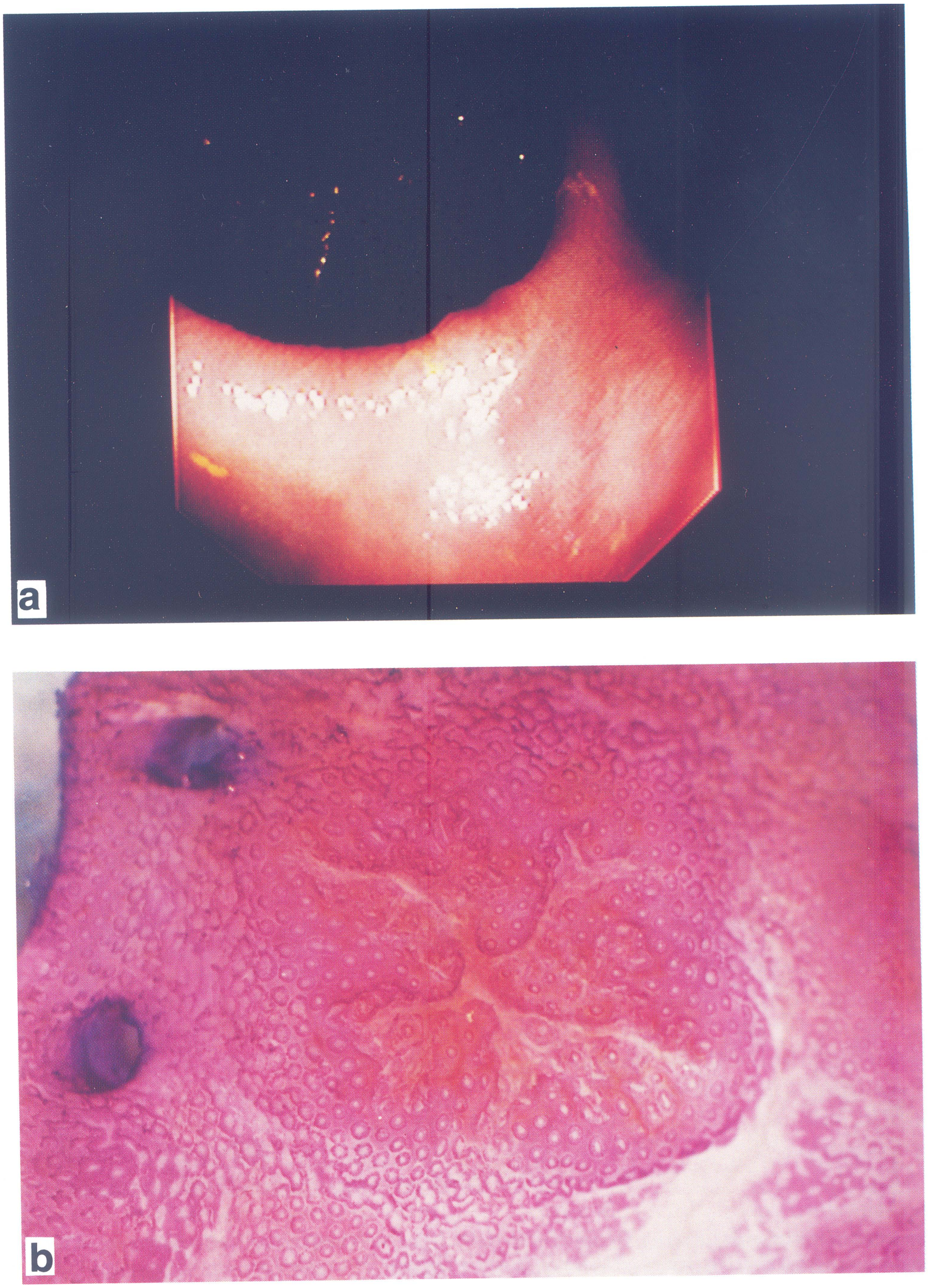

**Figure 3** (*Continued*)

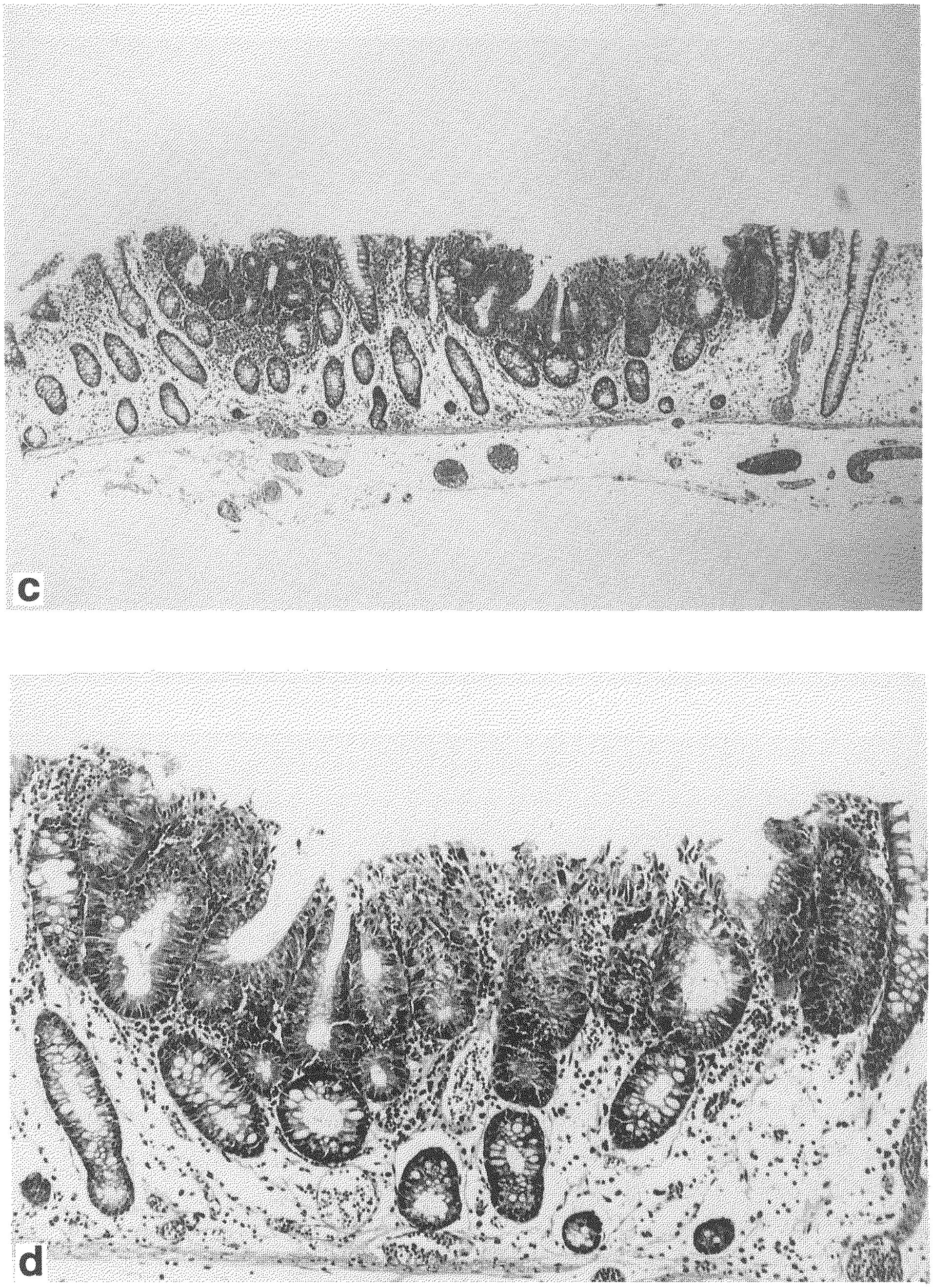

**Figure 3** A. Colonoscopic picture showing a small, hyperemic, depressive lesion with surrounding reactive elevation in the transverse colon. B. Dissecting microscopic view showed a slightly depressed lesion of ~4 mm in diameter. The surface property was mainly IIIS type. Its margin was irregular and surrounded by hyperplastic tubules. The marginal zone property was classified as B type. C. Cross-section of Figure 3B. The area of neoplastic ducts was slightly more depressed than the normal surrounding mucosa. The macroscopic type was classified as IIc + IIa. D. Histological picture at low magnification. The tubular density of the lesion was 78.97%, nuclear area was 37.03 $\mu$ $m^2$, and nuclear shortest/longest diameter ratio was 2.37. The atypia index obtained from the discriminant equation was +0.986. This lesion was classified into the HAI group. The histopathological diagnosis was adenoma with severe atypia.

both of these lesions were found to have higher variability in nuclear polarity than in nuclear size. It was therefore concluded that the pathological diagnosis of these lesions as highly atypical adenomas was based on nuclear polarity in addition to nuclear size and shape.

The diagnostic criteria for colonic cancer proposed by Watanabe et al.[5] globally evaluates cells based on nuclear staining property, polarity, and nucleolar shape in addition to nuclear size and shape. Therefore, the nuclear atypia index presented here may not be flawless. However, because the discriminant equation was very accurate in the control group and because all lesions pathologically evaluated to be either carcinomas or highly atypical adenomas were classified into the HAI group, nuclear size and shape are regarded as the most important indices of atypia. The results derived using these indices did reflect the degree of atypia and did not negatively affect the accuracy of diagnosis.

There was no relationship between macroscopic type and HAI/LAI group classification obtained by the discriminant equation. Correlations were investigated for all the measured variables including duct density, and for both superficial elevated type lesions (IIa) and mainly depressed lesions (IIc, IIc + IIa). A significant difference between these lesion types was detected only for the longest nuclear diameter and no clinically meaningful data were obtained. The fact that many lesions with mild atypia were found among IIc and IIc + IIa lesions demonstrates that depressed type adenomas are not at all rare.

Since the IIIS and IIIL classifications of Kudo et al.[6] are often intermingled at the tubule orifice, lesions in the study group were divided into two subgroups depending on which type was more dominant. Many reports claim that atypia is higher for lesions showing IIIS than for those showing IIIL.[6,7,18,19] However, a significant difference was not detected in this study, although the number of IIIL-dominant lesions tended to be lower in the HAI group. The relationship between various variables, including tubular density, and IIIS and IIIL classifications was studied, but there was no significant correlation. Generally, IIIL and IIIS are most commonly seen in protruded type lesions and superficial depressed type lesions, respectively, but all depressed type lesions (IIc and IIc + IIa) in this study were IIIS dominant. Likewise, for IIa lesions, 27 were IIIS dominant (79.4%) and 7 were IIIL dominant (20.6%), indicating that superficial type lesions tended to be IIIS. These classifications refer to specific histological characteristics of the tubule orifice in colonic tumors. Differences between these types may be associated with lesion morphology and may not be directly related to either tubular density (i.e., structural atypia) or nuclear dimensions (i.e., cellular atypia).

Comparison of marginal zone properties between the HAI group and the LAI group revealed a significantly higher frequency of a B-type marginal zone in the HAI group. Therefore, it is highly likely that lesions with high nuclear atypia present with a B-type marginal zone. To investigate which variables are related to marginal zone properties, lesions showing A-type (A group) were compared with lesions showing B-type (B group) in terms of nuclear area, longest diameter, shortest diameter, longest/shortest diameter ratio, and shape index, as well as tubular density. A highly significant relationship was obtained for tubular density only and not for any of the nuclear dimensions. Therefore, marginal zone properties were more closely related to tubular density than to nuclear shape. Since many lesions with high tubular density were included in the HAI group, many lesions in the HAI group probably had B-type marginal zone properties.

Structural atypia is evaluated based primarily on features such as a back-to-back arrangement, tubule asymmetry, and increased numbers of tubules. It is difficult to directly quantify these features to enhance objectivity. Ohkura and Nakamura[20] proposed tubular density as a global index of structural atypia.

Cellular atypia and structural atypia are regarded as two distinct entities that are not directly related. For highly differentiated carcinomas, cellular atypia is a more important diagnostic criterion than it is for undifferentiated carcinomas, and structural atypia, particularly tubular density, should serve as an auxiliary criterion for diagnosis. However, the fact that lesions with high nuclear atypia tended to show high tubular density in this study suggests that lesions that are very likely to become malignant have high tubular density.

The theory that the surface and margin zone properties of lesions refect tubular density to a greater extent than does nuclear shape has not been proven. A high density of neoplastic tubules applies pressure on the surrounding normal tissue and is thought to cause hyperplastic changes around lesions. Moreover, assuming that lesions with high tubular density tend to have high cellular (nuclear) atypia, it may be possible to identify lesions that are very likely to become malignant by closely inspecting their marginal zones endoscopically.

Since the atypia of depressed type lesions tends to be overevaluated on histopathological examination, the rate of false-positive diagnoses of cancer may be high for depressed type lesions showing IIIS characteristics at the tubule orifice. However, lesions with high tubular density are likely to have a fine, dense pattern at the tubule orifice. Quantification of tubular density in a clinical setting may therefore improve the accuracy of endoscopic diagnosis.

### CONCLUSIONS

1. Nuclear size and shape of 30 clear-cut adenomas and 30 clear-cut carcinomas were measured by image analysis to derive a discriminant equation allowing for correct diagnosis based on the degree of atypia.

2. Fifty superficial type neoplastic lesions (longest diameter <10 mm) of the colon, obtained by endoscopic resection, were similarly studied histopathologically to quantify nuclear dimensions. These lesions were divided into a high-atypia index group and a low-atypia index group by the discriminant equation mentioned above. The marginal zone properties of lesions with high atypia were assessed using a dissecting microscope. These lesions showed a significantly higher frequency of hyperplasia at the tubule orifice.

3. Tubular density tended to be higher in the high-atypia index group.

4. The marginal zone properties of lesions were more closely correlated to the tubular density than to nuclear dimensions.

5. The marginal zone properties of lesions were related to the tubular density. Since lesions with high nuclear atypia tended to have high tubular density, observation of the marginal zone properties of lesions may be useful in diagnosing lesions with severe atypia.

### ACKNOWLEDGMENTS

I am grateful to Prof. Tetsu Yamaguchi, Prof. Yoshihiro Sakai, and Dr. Sumio Fujinuma, Lecturer, of the Third Department of Internal Medicine, Toho University, for their guidance in this study and review of the manuscript. I also sincerely appreciate the help and cooperation of colleagues in the Department of Pharmacology and the Third Department of Internal Medicine, Toho University.

### REFERENCES

1. Kudoh S, Kusaka H, Kimata H, et al. The diagnosis and treatment of colorectal sm cancer. Stomach Intest 1991;26:764–775.
2. Igarashi M, Katsumata T, Mitomi H, et al. Treatment for colorectal sm cancer. Stomach Intest 1991;26:857–867.
3. Arima N, Toyonaga A, Tsuruta O, et al. Endoscopic diagnosis of the invasion depth of early colonic cancers. Endosc Dig 1992;4:1333–1342.
4. Nakamura K. The structure of colon cancer. Tokyo: Igakushoin, 1989:37–72.
5. Watanabe H, Ajioka Y, Yamaguchi M, et al. Relationship of colorectal adenoma and colorectal carcinoma—Based on the author's diagnostic criteria. Stomach Intest 1991;26:253–259.
6. Kudo S, Koji M, Takano Y, et al. Detection of colorectal minute cancer. Stomach Intest 1990;25:801–812.
7. Tada S, Iida M, Yao T, et al. Stereomicroscopic findings of adenoma and early cancer in the colorectum. Stomach Intest 1992;27:949–961.
8. Ajioka Y, Watanabe E, Chida T, et al. Pathological characteristics of small superficial type epithelial neoplasia of the large intestine with special reference to its macroscopic criteria. Stomach Intest 1990;25:837–846.
9. Nagasako K. Criteria of macroscopic feature of small colorectal carcinomas of superficial type (type II). Stomach Intest 1990;25:870–871.
10. Ohara T. Criteria of macroscopic feature of small colorectal carcinomas of superficial type (type II). Stomach Intest 1990;25:868–869.
11. Mutoh T. Criteria of macroscopic feature of small colorectal carcinomas of superficial type (type II). Stomach Intest 1990;25:870–871.
12. Shimoda T. Criteria of macroscopic feature of small colorectal carcinomas of superficial type (type II). Stomach Intest 1990;25:872–873.
13. Nagasako K. The significance of electronic endoscopy in the diagnosis of minute carcinomas of the colon. Endosc Dig 1990;2:997–1004.
14. Nakamura K, Shibuya S, Nishizawa M, et al. Adenoma-carcinoma sequence of colorectal carcinomas analysed by use of objective indices of grade of atypicality, and their growing processes in early phase. Stomach Intest 1985;20:877–888.
15. Ajioka Y, Watanabe H, Katagiri K, et al. Histological grade of atypia and development of the early colorectal cancer. Gastroenterol Surg 1992;15:1321–1328.
16. Morson BC, Dawson IMP. Gastrointestinal pathology, 2nd ed. Oxford: Blackwell Scientific, 1979.
17. Shimoda T, Ikegami M, Tei H, et al. Pathological study of early colorectal carcinoma. Stomach Intest 1987;22:967–976.
18. Toyonaga A, Arima N, Tsuruta O, et al. Diagnosis of depressed type early colorectal cancers from the endoscopic and pathological point of view. Stomach Intest 1992;27:911–923.
19. Hirata I, Morikawa H, Sugimoto K, et al. An issue concerning endoscopic diagnosis of superficial (type II) colorectal epithelial neoplasm. Stomach Intest 1992;27:903–909.
20. Ohkura Y, Nakamura K. Histopathological study on the histogenesis of colorectal carcinoma. Stomach Intest 1987;22:431–441.

